# Supplementary material for: Defect-Mediated Energy Transfer Mechanism by Modulating Lattice Occupancy of Alkali Ions for the Optimization of Upconversion Luminescence
Source: Nanomaterials (Basel). 2024 Dec 7;14(23):1969. doi: 10.3390/nano14231969 (PMC11643515; doi:10.3390/nano14231969)
Supplement: Supplementary file 1 [file nanomaterials-14-01969-s001.zip › nanomaterials-3324009-supplementary.pdf]

Supplementary Materials

Defect-Mediated Energy Transfer Mechanism by Modulating Lattice Occupancy of Alkali Ions for the Optimization of Upconversion Luminescence

Rongyao Gao [1], Yuqian Li [1], Yuhang Zhang [1], Limin Fu [1,*] and Luoyuan Li [2,*]

[1] Laboratory of Advanced Light Conversion Materials and Biophotonics, School of Chemistry and Life Resource, Renmin University of China, Beijing 100872, China; rygao@ruc.edu.cn (R.G.); liyuqian@ruc.edu.cn (Y.L.); zhangyuhang@ruc.edu.cn (Y.Z.)

[2] The Eighth Affiliated Hospital, Sun Yat-sen University, Shenzhen 518033, China

* Correspondence: lmfu@ruc.edu.cn (L.F.); lily98@mail.sysu.edu.cn (L.L.)

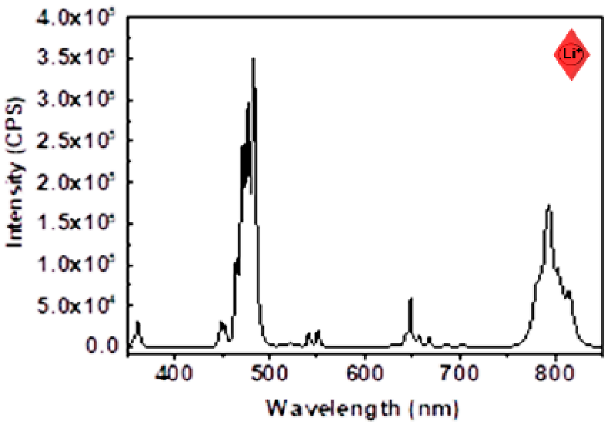

Figure S1. The steady-state PL luminescence spectra in vis-NIR regions of $LiLuF_4$:Yb, Tm nanoparticles under 980 nm(500 mW/cm^2) laser irradiation.

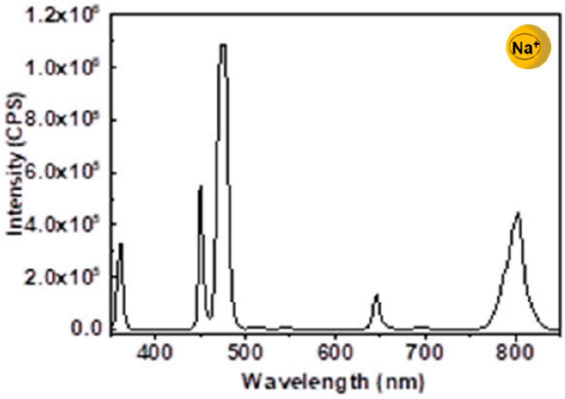

Figure S2. The steady-state PL luminescence spectra in vis-NIR regions of $NaLuF_4$:Yb, Tm nanoparticles under 980 nm(500 mW/cm^2) laser irradiation.

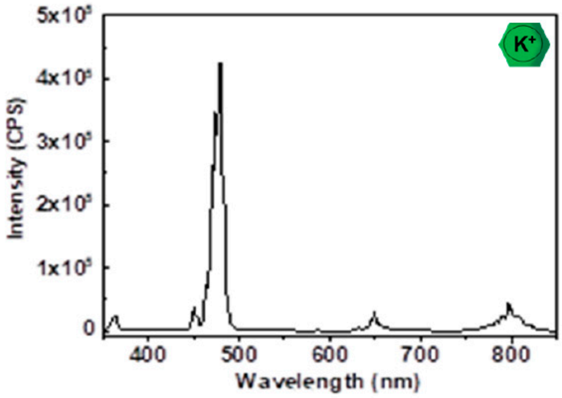

Figure S3. The steady-state PL luminescence spectra in vis-NIR regions of KLu_2F_7:Yb, Tm nanoparticles under 980 nm(500 mW/cm^2) laser irradiation.

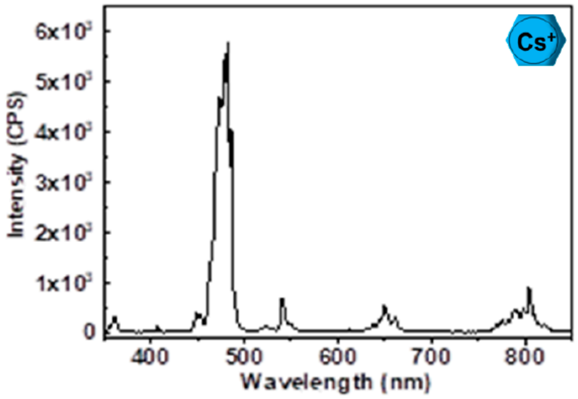

Figure S4. The steady-state PL luminescence spectra in vis-NIR regions of $CsLu_2F_7$:Yb, Tm nanoparticles under 980 nm(500 mW/cm^2) laser irradiation.

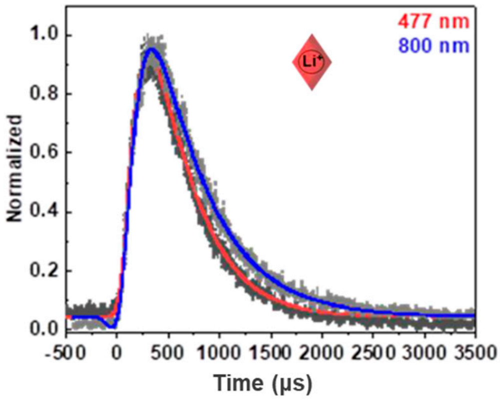

Figure S5. The kinetic curves and fitting lines of the $^1G_4$ (477 nm, black dots) and $^3H_4$ (800 nm, gray dots) levels of Tm^{3+} in the $LiLuF_4$ nanoparticles.

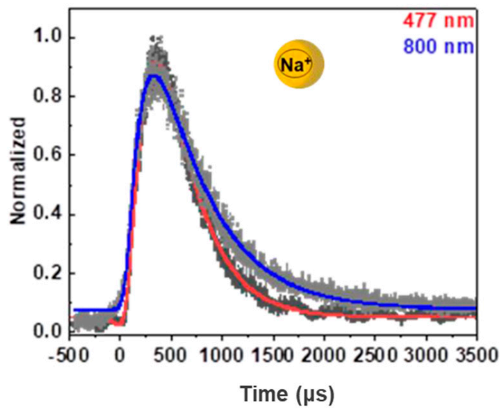

Figure S6. The kinetic curves and fitting lines of the $^1G_4$ (477 nm, black dots) and $^3H_4$ (800 nm, gray dots) levels of Tm^{3+} in the $NaLuF_4$ nanoparticles.

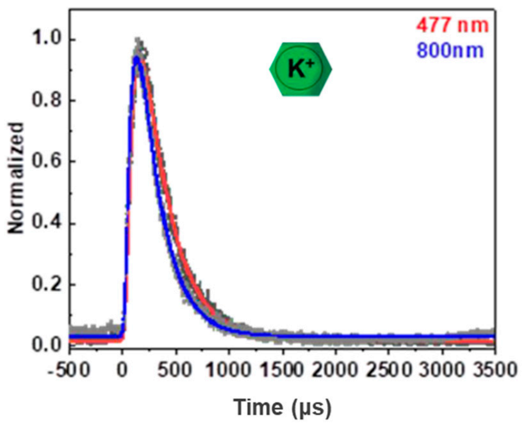

Figure S7. The kinetic curves and fitting lines of the $^1G_4$ (477 nm, black dots) and $^3H_4$ (800 nm, gray dots) levels of Tm^{3+} in the KLu_2F_7 nanoparticles.

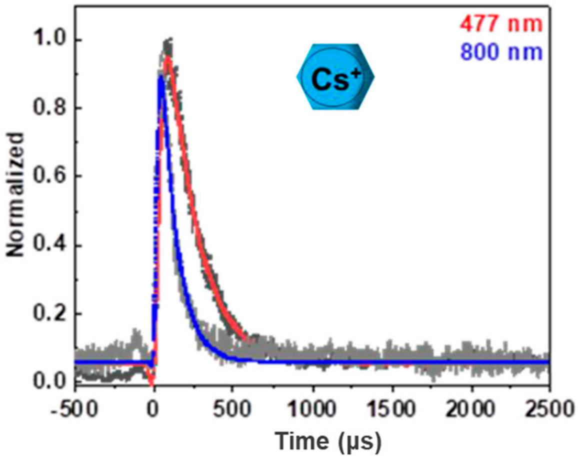

Figure S8. The kinetic curves and fitting lines of the $^1G_4$ (477 nm, black dots) and $^3H_4$ (800 nm, gray dots) levels of Tm^{3+} in the $CsLu_2F_7$ nanoparticles.

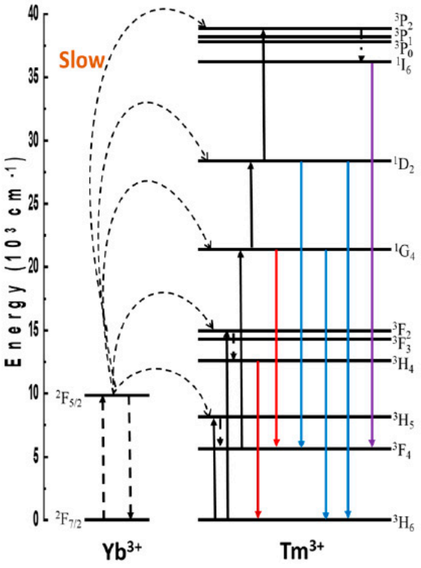

Figure S9. Energy transfer mechanisms of $MLuF_4$:Yb^{3+}, Tm^{3+} (M: Li, Na) nanoparticles.

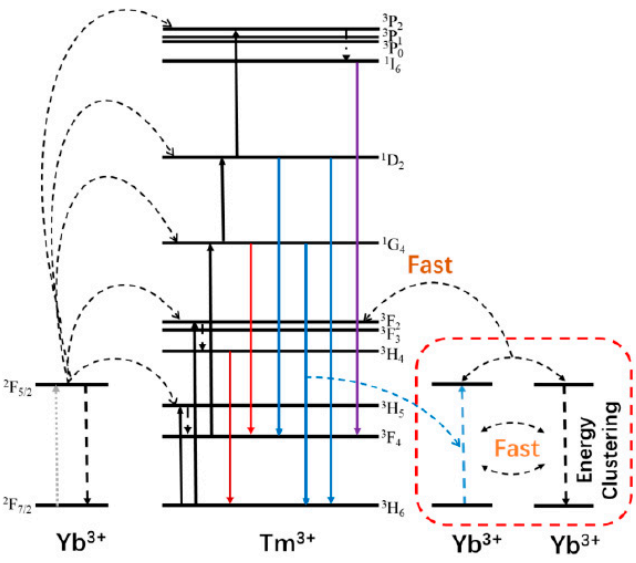

Figure S10. Energy transfer mechanisms of MLu_2F_7:Yb^{3+}, Tm^{3+} (M: K, Cs) nanoparticles.
